# Enzymatically active Rho and Rac small-GTPases are involved in the establishment of the vacuolar membrane after *Toxoplasma gondii* invasion of host cells

**DOI:** 10.1186/1471-2180-13-125

**Published:** 2013-05-30

**Authors:** Ren-Hua Na, Guo-Hui Zhu, Ji-Xuan Luo, Xiao-Jing Meng, Liwang Cui, Hong-Juan Peng, Xiao-guang Chen, Julian Gomez-Cambronero

**Affiliations:** 1 Key Laboratory of Prevention and Control for Emerging Infectious Diseases of Guangdong Higher Institutes, Department of Pathogen Biology, School of Public Health and Tropical Medicine, Southern Medical University, Guangzhou, Guangdong 510515, China; 2 Department of Occupation Hygiene, School of Public Health and Tropical Medicine, Southern Medical University, Guangzhou, Guangdong, 510515, China; 3 Department of Entomology, Pennsylvania State University, 537 ASI Bldg, University Park, PA, 16802, USA; 4 Department of Biochemistry and Molecular Biology, Wright State University School of Medicine, 3640 Colonel Glenn Highway, Dayton, OH, 45435, USA

**Keywords:** *Toxoplasma gondii*, Parasitophorous vacuole membrane, RhoA, Rac1, GTPase, Accumulate, Activate

## Abstract

**Background:**

GTPases are the family of hydrolases that bind and hydrolyze guanosine triphosphate. The large Immunity-related GTPases and the small GTPase ADP-ribosylation factor-6 in host cells are known to accumulate on the parasitophorous vacuole membrane (PVM) of *Toxoplasma gondii* and play critical roles in this parasite infection, but these GTPases cannot explain the full extent of infection.

**Results:**

In this research, RhoA and Rac1 GTPases from the host cell were found to accumulate on the PVM regardless of the virulence of the *T. gondii* strains after *T. gondii* invasion, and this accumulation was dependent on their GTPase activity. The real-time micrography of *T. gondii* tachyzoites invading COS-7 cells overexpressing CFP-RhoA showed that this GTPase was recruited to the PVM at the very beginning of the invasion through the host cell membrane or from the cytosol. Host cell RhoA and Rac1 were also activated after *T. gondii* tachyzoites invasion, which was needed for host cell cytoskeleton reorganization to facilitate intracellular pathogens invasion. The decisive domains for the RhoA accumulation on the PVM included the GTP/Mg^2+^ binding site, the mDia effector interaction site, the G1 box, the G2 box and the G5 box, respectively, which were related to the binding of GTP for enzymatic activity and mDia for the regulation of microtubules. The recruited CFP-RhoA on the PVM could not be activated by epithelial growth factor (EGF) and no translocation was observed, unlike the unassociated RhoA in the host cell cytosol that migrated to the cell membrane towards the EGF activation spot. This result supported the hypothesis that the recruited RhoA or Rac1 on the PVM were in the GTP-bound active form. Wild-type RhoA or Rac1 overexpressed cells had almost the same infection rates by *T. gondii* as the mock-treated cells, while RhoA-N19 or Rac1-N17 transfected cells and RhoA, Rac1 or RhoA + Rac1 siRNA-treated cells showed significantly diminished infection rates compared to mock cells.

**Conclusions:**

The accumulation of the RhoA and Rac1 on the PVM and the requisite of their normal GTPase activity for efficient invasion implied their involvement and function in *T. gondii* invasion.

## Background

*Toxoplasma gondii* is an intracellular protozoan that infects many types of nucleated cells. It is estimated that approximately one-third of the world’s population is chronically infected with tissue cysts of this parasite [1]. Humans may be infected through ingestion of uncooked or under-cooked meat of intermediate hosts or the oocysts excreted by the definitive host, *Felis catus*. Ingested bradyzoites and tachyzoites invade host cells and cause acute infection. In humans, *T. gondii* infections may cause disseminating damage to the brain, eyes, lymph nodes and even death in some immunocompromised individuals [2]. In pregnant women, this parasite can be transmitted to the fetus, resulting in tissue destruction, as well as developmental defects of the fetus or newborn [2]. In immunocompetent hosts, tachyzoites are converted into bradyzoites quickly, and a lifelong chronic infection is established.

The molecular mechanism of host cell invasion by *T. gondii* has been extensively investigated [2]. During invasion, a *T. gondii* tachyzoite attaches to the host cell membrane and forms a moving junction (MJ) between the tachyzoite and the host cell membrane by releasing microneme proteins (MIC) and rhoptry neck proteins (RON) at the interface of the tachyzoite-host cell surface. Afterwards, the tachyzoite membrane and the host cell membrane remain in contact so that the MJ moves along the parasite’s surface until the parasitophorous vacuole (PV) is finally formed [3,4]. The MJ works as a sieve to exclude many of the host transmembrane proteins but retains GPI-anchored or raft-associated multipass transmembrane proteins on the PV membrane (PVM) [3,4]. PVM is a non-fusogenic compartment that is resistant to acidification by the endosome-lysosomal system of the host cell, since most of the PVM is derived from the host cell membrane and the transmembrane proteins, which are involved in fusion with lysosomes and are excluded from the PVM [3-5]. During penetration, the parasite injects many rhoptry proteins including ROP2 into the host cell cytosol, which appear as small satellite vesicles and eventually fuse with the PVM [6]. After invasion, the parasite further modifies the PVM by inserting novel proteins secreted by the rhoptries and the dense granules [7,8]. After formation, the PVM closely associates with host mitochondria and endoplasmic reticulum (ER) and migrates towards the nucleus using the host microtubule network [9].

GTPases are a large group of enzymes that bind GTP (guanine triphosphate) and catalyze the hydrolysis of GTP to GDP (guanine diphosphate) in the presence of a Mg^2+^ ion. They then undergo conformational changes to release GDP, and thus, cycle between a GTP-bound active form and a GDP-bound inactive form [10]. Immune related GTPases (IRG) are large GTPases containing a Ras-like G domain and a helical domain combining N- and C-terminal elements [11], whereas small GTPases are monomeric GTPases with a molecular weight of 21 kDa and composed of at least five families: Ras, Rho, Rab, Sar1/Arf and Ran, which exist in eukaryotes from yeast to humans [12]. The Rho subfamily is further divided into RhoA, Rac and Cdc42, which regulates cytoskeleton reorganization and gene expression [13].

A group of interferon-inducible large GTPases (IRGs) and a small GTPase, ADP-ribosylation factor-6 (ARF6) of the host cell accumulate on the PVM of invading *T. gondii*[14,15]. IFN-γ-Inducible GTPase (Irga6) is a myristoylated IRG and contributes to resistance against *T. gondii* in mice. Irga6 is predominantly found in the GDP-bound state in interferon-induced, uninfected cells, but it does accumulate on the PVM after *Toxoplasma* infection and changes to the GTP-bound form. Accumulation of Irga6 on the *T. gondii* PVM is associated with vesiculation and ultimately disruption of the vacuolar membrane in a process that requires an intact GTP-binding domain [16]. ARF6 is recruited to the PVM of *T. gondii* RH strain and plays an important role in the parasite cell invasion with activation of PI3-kinase and recruitment of PIP_2_ and PIP_3_ to the PVM of *T. gondii*[14]. The significance of some GTPases in the *Toxoplasma* invasion process has prompted us to further investigate whether other members of the small GTPases are also involved in host cell invasion.

## Methods

### Ethics statement

KM white mice were purchased from the Laboratory Animal Center of Southern Medical University. Mice were housed in the facility at the School of Public Health and Tropical Medicine according to the guidelines for laboratory animals approved by Guangdong Laboratory Animals Monitoring Institute. This research does not involve human participants, and it was approved by the Institutional Ethics Review Board of Southern Medical University.

### Plasmids construction and site mutation

The cDNAs of RhoA-N19 and Rac1-N17 were generous gifts from Dr. Wei Li (University of Southern California, Los Angeles, CA). These cDNAs were amplified with PCR to incorporate *Sal*I and *Sac*II restriction sites on 5`- and 3`-ends, respectively, for cloning and was subcloned into the pECFP-N1 vector. The recombinant plasmids were verified by DNA sequencing. pECFP‒RhoA WT and pECFP‒Rac1 WT were site-mutated from pECFP-RhoA-N19 and pECFP-Rac1-N17, respectively. The 10 aa sequentially truncated RhoA were generated with QuikChange II Site-Directed Mutagenesis Kits from Stratagene. All the mutation primers are shown in Table 1.

**Table 1:** The mutation primers used to generate all the mutants

**Mutants**	**Template**	**Mutation primers**
M1 (RhoA^Δ1-10^)	pECFP-RhoA WT (1-10aa deleted)	forward: 5’-GTCGACGATTTCGACGTTGGTGATGGAGCC-3’
		reverse: 5’-GGCTCCATCACCAACGTCGAAATCGTCGAC-3’,
M2 (RhoA^Δ11-20^)	pECFP-RhoA WT (11-20aa deleted)	Forward: 5’-CGGAAGAAACTGGTGATTTTGCTCATAGTTAACAGC-3’
		Reverse: 5’- GCTGTTAACTATGAGCAAAATCACCAGTTTCTTCCG-3’
M3 (RhoA^Δ21-30^)	pECFP-RhoA WT (21-30aa deleted)	Forward: 5’-CTGTGGAAAGACATGCCCAGAGGTGTATGTGC-3’
		Reverse: 5’- GCACATACACCTCTGGGCATGTCTTTCCACAG-3’
M4 (RhoA^Δ31-40^)	pECFP-RhoA WT (31-40aa deleted)	Forward: 5’- GCGAGGACCAGTTCAACTATGTGGCAG-3’
		Reverse: 5’- CTGCCACATAGTTGAACTGGTCCTCGC-3’
M5 (RhoA^Δ41-50^)	pECFP-RhoA WT (41-50aa deleted)	Forward: 5’-GCCCACAGTGTTTGAGAAGCAGGTAGAGTTGG-3’
		Reverse: 5’- CCAACTCTACCTGCTTCTCAAACACTGTGGGC-3’
M6 (RhoA^Δ51-60^)	pECFP-RhoA WT (51-60aa deleted)	Mutant 6 Forward: 5’-CGAGGTGGATGGAGCTGGGCTGGAAG-3’
		Mutant 6 Reverse: 5’- CTTCCAGCCCAGCTCCATCCACCTCG -3’
M7 (RhoA^Δ61-70^)	pECFP-RhoA WT (61-70aa deleted)	Forward: 5’-CTTTGTGGGACACACCCCTCTCCTACCC-3’
		Reverse: 5’-GGGTAGGAGAGGGGTGTGTCCCACAAAG-3’
M8 (RhoA^Δ71-80^)	pECFP-RhoA WT (71-80aa deleted)	Forward: 5’-GATTATGATCGCCTGAGGCTGATGTGTTTTTCCATC-3’
		Reverse: 5’- GATGGAAAAACACATCAGCCTCAGGCGATCATAATC-3’
M9 (RhoA^Δ81-90^)	pECFP-RhoA WT (81-90aa deleted)	Forward: 5’-CCCAGATACCGATGTTATAAGTTTAGAAAACATCCCAG-3’
		Reverse: 5’-CTGGGATGTTTTCTAAACTTATAACATCGGTATCTGGG-3’
M10 (RhoA^Δ91-100^)	pECFP-RhoA WT (91-100aa deleted)	Forward: 5’-CGACAGCCCTGATCCAGAAGTCAAGC-3’
		Reverse: 5’- GCTTGACTTCTGGATCAGGGCTGTCG-3’
M11 (RhoA^Δ101-110^)	pECFP-RhoA WT (101-110aa deleted)	Forward: 5’-CAGAAAAGTGGACCCCCATCATCCTGG-3’
		Reverse: 5’-CCAGGATGATGGGGGTCCACTTTTCTG-3’
M12 (RhoA^Δ111-120^)	pECFP-RhoA WT (111-120aa deleted)	Forward: 5’-CTGTCCCAACGTGCTTCGGAATGATG-3’
		Reverse: 5’- CATCATTCCGAAGCACGTTGGGACAG-3’
M13 (RhoA^Δ121-130^)	pECFP-RhoA WT (121-130aa deleted)	Forward: 5’-GTTGGGAATAAGAAGGATCTAGCCAAGATGAAGCAG-3’
		Reverse: 5’-CTGCTTCATCTTGGCTAGATCCTTCTTATTCCCAAC-3’
M14 (RhoA^Δ131-140^)	pECFP-RhoA WT (131-140aa deleted)	Forward: 5’-CACACAAGGCGGGAGCCTGAAGAAGGCAG-3’
		Reverse: 5’-CTGCCTTCTTCAGGCTCCCGCCTTGTGTG-3
M15 (RhoA^Δ141-150^)	pECFP-RhoA WT (141-150aa deleted)	Forward: 5’-GGAGCCGGTGAAAATTGGCGCTTTTG-3’
		Reverse: 5’- CAAAAGCGCCAATTTTCACCGGCTCC-3’
M16 (RhoA^Δ151-160^)	pECFP-RhoA WT (151-160aa deleted)	Forward: 5’-GAGATATGGCAAACAGGGCAAAGACCAAAGATGG-3’
		Reverse: 5’- CCATCTTTGGTCTTTGCCCTGTTTGCCATATCTC-3’
M17 (RhoA^Δ161-170^)	pECFP-RhoA WT (161-170aa deleted)	Forward: 5’-GTGCATGGAGTGTTCATTTGAAATGGCTACG-3’
		Reverse: 5’- CGTAGCCATTTCAAATGAACACTCCATGCAC-3’
M18 (RhoA^Δ171-180^)	pECFP-RhoA WT (171-180aa deleted)	Forward: 5’-GGAGTGAGAGAGGTTGCTAGACGTGGGAAG-3’
		Reverse: 5’- CTTCCCACGTCTAGCAACCTCTCTCACTCC-3’
M19 (RhoA^Δ181-192^)	pECFP-RhoA WT (181-192aa deleted)	Forward: 5’-GAGCTGCTCTGCAACTTGTCTTGCCGCG-3’
		Reverse: 5’- CGCGGCAAGACAAGTTGCAGAGCAGCTC-3’

### Animals, toxoplasma gondii strains and cell lines

Pathogens free (SPF) KM mice were bought from the Animal Institute of Southern Medical University (Guangdong Province, China). *T. gondii* RH and Pru strain were generous gift from Dr. Xi-Mei Zhan in the School of Medicine of Sun Yat-sen University. The COS-7 cell line was purchased from ATCC and the human bronchial epithelial (16-HBE) cell line was purchased from Shanghai Fuxiang Biotechnology Limited Company. Each cell line was grown in DMEM (Gibco) containing 10% (v/v) NCS (New born calf serum, Gibco) at 5% CO_2_ and 37°C. For fluorescence microscopy and *T. gondii* infection rate counting experiments, COS-7 cells were grown on coverslips in the wells of 6-well plates (Corning). 16-HBE cells were used for RNAi and endogenous RhoA and Rac1 immunofluorescence experiments.

### Toxoplasma gondii infection

#### RH strain tachyzoites

Tachyzoites of the RH strain of *T. gondii* were harvested from the peritoneal cavities of KM mice which were inoculated with 100–200 tachyzoites per mouse three days before intraperitoneal injection.

#### Pru strain tachyzoites

*T. gondii* Pru strain chronically infected mice (intra-gastric inoculation with Pru cysts for more than 45 days) were euthanized and the brains were used for cysts separation. The brain homogenates were washed 2 times with Phosphate Buffered Saline (PBS). Lymphocytes separation medium (Sigma-Aldrich, 10771) was used to separate the lymphocyte from the cysts, and the cysts were collected from the bottom of the separation phases. The cysts were inoculated into peritoneal cavities of KM mice; the tachyzoites of Pru strain were then harvested from the ascites ten days post-infection.

#### Tachyzoites infection of cells

The harvested ascites were centrifuged for 5 min at room temperature at 3000 × g and quickly resuspended in DMEM complete medium. Cells transfected with plasmids or treated with siRNA for 48 h were infected with 1 × 10^5^ *T. gondii* RH or Pru strain tachyzoites per well for 2 hr.

#### Transfection of plasmid DNA and short interference RNA (siRNA)

COS-7 cells were seeded in the 6-well plates and reached 70% confluence. Three μg of plasmid DNA per well were used for transfection with Lipofectamine™ LTX and plus reagent (invitrogen). Stealth double-stranded RhoA siRNA, and Rac1 siRNA and negative control (Neg Ctrl) siRNA were synthesized by Invitrogen (Carlsbad, CA, USA). SiRNA transfection was performed 24 hr after 16-HBE cells were seeded in the wells and reached 85% confluence. One hundred nmol of RhoA or Rac1 siRNA were used to silence RhoA or Rac1 separately and 100 nmol RhoA plus 100 nmol Rac1 siRNA were used for double silencing for transfection with Lipofectamine™ RNAi MAX (invitrogen). Mock transfection only contained transfection reagents.

#### Detection of the RNAi efficiency

The RNA interference (RNAi) efficiency was checked by Western-blot. The cells were harvested and lysed with RIPA lysis buffer (Thermo Scientific). One hundred μg of total proteins per well were loaded onto a SDS-PAGE gel and then transferred to a PVDF membrane for western blot detection.

#### GST pull down assay to detect the activation of RhoA and Rac1

16-HBE cells were cultured in six T-75 flasks to reach 100% confluency. Three flasks of cells were infected with *T. gondii* tachyzoites at a multiplicity of infection (MOI) of 10. The other three flasks of cells were kept as uninfected control (mock). At 3 hr post-infection, the medium from mock and infected flasks was aspirated and cells were trypsinized. Mock and infected cells were lysed in RIPA lysis buffer (Thermo Scientific) with ultrasonication. For negative control, 150 μg (600 μl) of the infected cell extract were aliquoted into two experimental tubes; 60 μl of loading buffer were added to each tube to a final concentration of 15 mM EDTA; 6 μl of GDP were added to these two tubes to a final concentration of 1.0 mM GDP and the tubes were incubated at room temperature for 15 min; the reaction was stopped by adding 60 μl of stopping buffer to each tube to a final concentration of 60 mM MgCl_2_.

The negative control cell lysate incubated with GDP, and 150 μg (600 μl) total protein from the lysate of infected, uninfected cells and *T. gondii* tachyzoites were added to 30 μg reconstituted GST-tagged Rhotekin-RBD protein on colored agarose beads for RhoA (Cytoskeleton Inc) or GST-tagged PAK-PBD protein bound colored agarose beads for Rac (Cytoskeleton Inc) respectively, and incubated at 4°C with rotating overnight. The beads were washed with PBS for 3 times. 25 μl protein loading buffer was added to each group of beads and boiled for 5 min then sediment at 12000 rpm for 1 min, the supernatant was used for SDS-PAGE. At the same time, 150 μg of total protein from the lysates of infected and uninfected cells and the *T. gondii* tachyzoites were used for SDS –PAGE, and actin in each group was detected via western-blot and used as the equal protein loading control for the GST pull down assay.

#### Western-blot reagents

Primary antibodies: monoclonal rabbit anti-human RhoA antibody (Cell Signaling) and polyclonal rabbit anti-human Rac1 antibody (Abcam) were used in 1:1000 dilutions; β-actin was detected for loading control with monoclonal mouse anti-human anti-actin antibody (Cell Signaling) in 1:5000 dilutions. Secondary antibody: polyclonal sheep anti-mouse IgG-HRP antibody (Abcam) and polyclonal goat anti-rabbit IgG-HRP antibody (Abcam) were used in 1:3000 dilutions. ECL Western Blotting detection reagent was purchased from Pierce.

#### Immunofluorescence for endogenous RhoA and Rac1 after T. gondii infection

16-HBE cells were grown on coverslips to 80% confluence. The cells were infected with *T. gondii* RH tachyzoites. Two hr post-infection the unrecruited parasites were washed away with PBS. The cells were fixed with polyformaldehyde for 30 min and permeablized with Triton X-100 and blocked with 5% BSA in PBS. The cells were incubated with the primary antibody at 4°C overnight. The coverslips were washed 3 times with PBST, 5 min each wash, and then FITC conjugated secondary antibody was added to the coverslips and incubated for 1 hr at room temperature. The coverslips were washed 3 times with PBST, 5 min each wash, and stained with 10 nM DAPI (Sigma) for 10 min at room temperature, then washed three times with PBS (5 min each wash) with slight shaking. The coverslips were rinsed with double distilled water and air-dried. The coverslips were mounted and ready for fluorescence microscopy.

Primary antibodies of monoclonal rabbit anti-human RhoA antibody (Cell Signaling) and polyclonal rabbit anti- human Rac1 antibody (Abcam) were used in 1:100 dilutions. Secondary antibody of goat anti-Rabbit IgG-FITC (Abcam) was used in 1:500 dilutions.

#### Fluorescence microscopy for overexpressed CFP tagged RhoA and Rac1

COS-7 cells grown on the coverslips were transfected with the CFP-tagged RhoA and Rac1 plasmids for 48 hr, and then infected with tachyzoites for 2 hr. Washed three times with PBS, the cells were fixed in 4% polyformaldehyde for 30 min. After aspiration, cells were stained with 10 nM DAPI (Sigma) for 10 min at room temperature, then washed three times with PBS (5 min each wash) with slight shaking. The coverslips were rinsed with double distilled water, air dried and mounted for fluorescence microscopy.

#### Infection rate counting and statistical analysis

16-HBE cells, mock or transfected with Neg Ctrl siRNA, RhoA siRNA, Rac1 siRNA, and RhoA + Rac1 siRNA were infected with 1 × 10^5^ *T. gondii* RH tachyzoites per well for 3 hr. The cells were washed 3 times with PBS. After aspiration, cells were stained with Giemsa stain (Sigma) for 10 min, and then washed three times with PBS (5 min each wash) with slight shaking. The coverslips were rinsed with double distilled water and air dried. The COS-7 cells overexpressing the CFP chimeras and the mock cells were also infected with 1 × 10^5^ *T. gondii* RH tachyzoites per well for 2 hr. The COS-7 mock cells were stained with Giemsa stain as above mentioned. The CFP chimeras overexpressed in COS-7 cells do not need staining. The coverslips were rinsed with double distilled water and air dried.

The coverslips were mounted and ready for infection rate counting. For Giemsa stained cells, infection rate was the percentage of *T. gondii* tachyzoites infected cells among 100 cells randomly selected. In the CFP-tagged overexpressed group, the infection rate was presented as the percentage of those tachyzoites infected fluorescent cells among 100 fluorescent cells randomly selected. The infection rate experiment was performed in triplicate. The infection rates were divided by the mean infection rate of the mock group, and the percentages were used to compare the infection rate difference between different groups.

All data were shown as the mean ± S.E.M (Standard Error of Mean) for three separate experiments. The difference was analyzed based on One-way ANOVA and LSD test by SPSS software package. The statistical significance was defined as *P* < 0.01.

#### EGF activation of cytosol Rho GTPases in COS-7 cells and the translocalization observation

COS-7 cells transfected with pECFP-RhoA WT were starved overnight in DMEM medium without serum. On the second day, the cells were infected with RH tachyzoites for 2 hr. The media was aspirated after infection and cells were washed three times with PBS. For epidermal growth factor (EGF, Sigma, E9644 ) activation, 300 μl DMEM medium without serum was added to each well, 2 μl of 100 ng/μl EGF was added to one corner of the coverslips. The cells were fixed with paraformaldehyde 5 min after activation. The fixed cells were stained with DAPI for DNA visualization, and then washed 3 times with PBS (5 min each wash) with slight shaking. The coverslips were rinsed with double distilled water and air dried. At this point, coverslips were ready for the observation of RhoA GTPases translocalization.

#### Real-time observation of RhoA GTPase recruited to the PVM following *T. gondii* tachyzoites invasion

COS-7 cells were grown on 2 cm confocal plates and transfected with 3 μg pECFP-N1-Rho A WT when cells reached 70% confluency. Forty-eight hr later *T. gondii* RH tachyzoites were used to infect these COS-7 cells. The confocal plate was incubated in the tray (with 5% CO_2_ at 37°C) and connected to the confocal fluorescence microscope (Olympus FluoView^®^ FV1000). The process of tachyzoites invading the host cell was visualized and pictures were taken automatically every 10 min.

## Results

### Accumulation of Rho and Rac GTPases on the PVM

**Figure 1:**
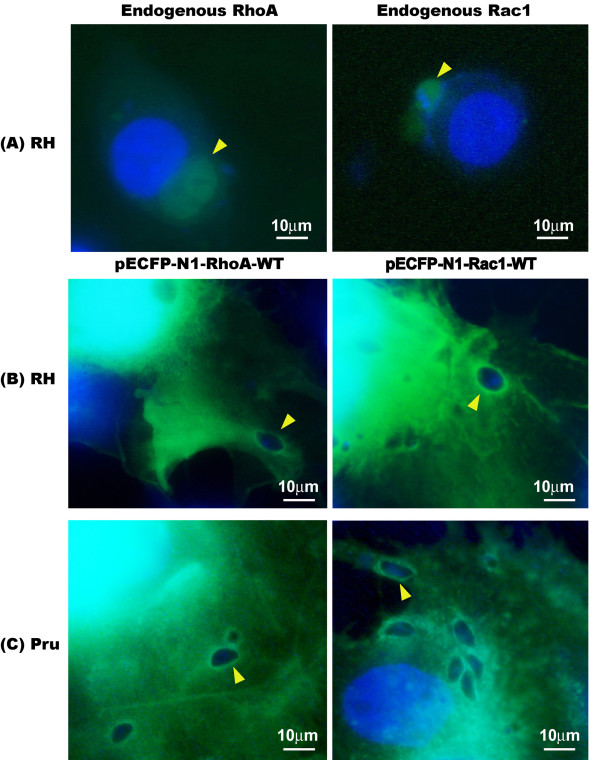
**The accumulation of Rho GTPases in the parasitophorous vacuole membrane (PVM) of *T. gondii *tachyzoites (1000×). **(**A**) The tachyzoites of *T. gondii *RH strain infected human 16-HBE cells were fixed with paraformaldehyde and permeablized with Triton X-100. The anti-RhoA and Rac1 primary antibodies were used to bind with the endogenous GTPases, then a FITC conjugated secondary antibody was used to bind with the primary antibodies. The endogenous RhoA and Rac1 accumulated on the PVM are visualized with a fluorescence microscope. (**B**-**C**) COS-7 cells were transfected with 3 μg of pECFP-N1-RhoA-WT and pECFP-N1-Rac1-WT, respectively. Forty-eight hr after transfection, these cells were infected with tachyzoites of *T. gondii *RH strain (**B**) or Pru strain (**C**). Regardless of the virulence of the tachyzoites used for infection, the overexpressed CFP-RhoA and CFP-Rac1 in host cells were recruited to the *T. gondii *PVM. Bars: 10 μm.

IRGs and Arf6 are members of large and small GTPase families, respectively, which accumulate on the PVM of *T. gondii* infected cells and play important roles during host cell invasion [14,15]. However, the presence of these two GTPases is insufficient to explain the whole spectrum of cell signaling during infection. To determine whether other GTPases, namely RhoA and Rac1 are also recruited to the PVM, the tachyzoites of *T. gondii* RH strain were used to infect human 16-HBE cells, and Rho and Rac1 were localized by indirect immunofluorescence assay (IFA) using anti-Rho and -Rac1 antibodies. IFA revealed significant accumulation of these two small GTPases on the PVM. To further verify this observation, CFP-tagged RhoA and Rac1 were overexpressed in COS-7 cells, and 48 hr post-transfection, cells were infected with different virulent strains of RH and Pru tachyzoites, respectively. Regardless of the virulence of the parasite strains used, RhoA and Rac1 were recruited to the PVM (Figure 1).

### Real-time observation of recruitment of RhoA GTPase to the PVM

**Figure 2:**
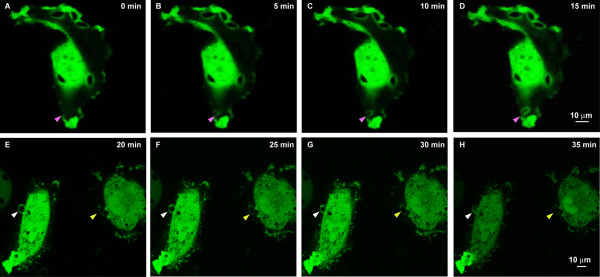
**The real-time observation of RhoA GTPase being recruited to the parasitophorous vacuole membrane (PVM) following *T. gondii *tachyzoites invasion (1000×). **(**A-F**) Starting from 0 min after the tachyzoites being added to the COS-7 cells transfected with pECFP-RhoA-WT, the invasion of tachyzoites into the host cell was visualized under a confocal microscope and pictures were taken at 5 min intervals. The CFP-tagged RhoA on the host cell membrane is recruited to the PVM at the same time as the tachyzoites started to invade the host cell (**A**, pink arrowhead). The accumulation of the RhoA to the PVM continued with the invasion of the tachyzoite into the host cell (**B**-**D**, pink arrowhead), until the whole tachyzoite was totally recruited into the host cell (**E**, white and yellow arrowhead). The loading of the RhoA GTPase onto the PVM continued after the tachyzoite was totally within the host cell, in this case, probably through the means of diffusion from the host cell cytosol (**E**-**H**, white and yellow arrowhead). The green fluorescence and the DIC images showing the observation of the invasion processes are provided in Additional file 1: Data S1 and Additional file 2: Data S2. Bar: 10 μm.

To follow the events of RhoA GTPase recruitment to the PVM, COS-7 cells transfected with pECFP-RhoA WT were infected with *T. gondii* RH tachyzoites. The real-time photographs were taken at 0 min post-infection and every 5 min thereafter using a confocal fluorescence microscope (Figure 2).

We found that the CFP-tagged RhoA was recruited to the PVM at the very beginning of the invasion, probably through retention of the RhoA GTPase on the host cell membrane to the PVM, and the accumulation of RhoA on the PVM continued with the recruitment of the tachyzoite until it totally invaded into the host cell (Figure 2A-D: pink arrowhead). However, a focal point of RhoA was not seen at the immediate point of invasion (Figure 2A). After the tachyzoite was completely inside the host cell, the fluorescence intensity continually increased with time, suggesting continuous accumulation of the RhoA GTPase on PVM probably by diffusion from the host cell cytosol (E-H, white and yellow arrowhead). The green fluorescence and the DIC images showing the invasion processes are provided in Additional file 1: Data S1 and Additional file 2: Data S2.

### The recruitment of Rho A and Rac1 GTPases into PVM is dependent on the GTPase activity

**Figure 3:**
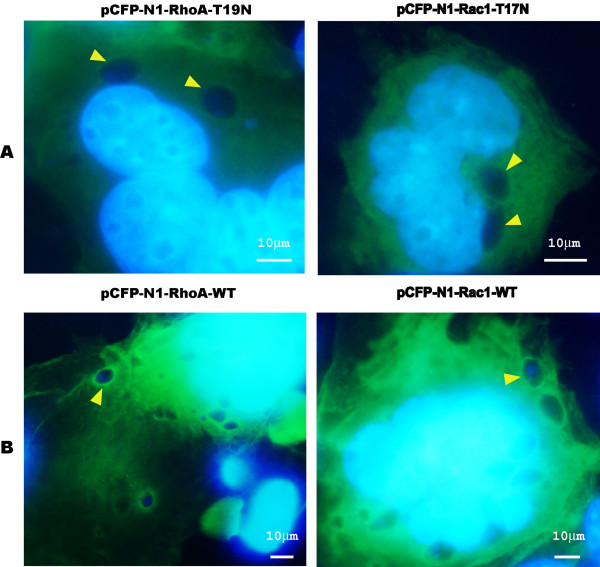
**The recruitment of RhoA and Rac1 GTPases into parasitophorous vacuole membrane (PVM) is dependent on the GTPase activity (1000×).** (**A**) The CFP-tagged dominant negative mutants RhoA N19 and Rac1 N17 were overexpressed in COS-7 cells and 48 hr post-transfection, the cells were infected with *T. gondii *RH tachyzoites. All of these mutant proteins did not accumulate on the PVM of *T. gondii* (arrowhead). (**B**) The CFP-tagged wild type RhoA and Rac1 were overexpressed in COS-7 cells and 48 hr post-transfection, the cells were infected with *T. gondii* RH tachyzoites. All of these wild-type proteins accumulated on the PVM of *T. gondii *(arrowhead). Bars: 10 μm.

We next investigated if intact GTPase activity was required for PVM recruitment. We used dominant negative mutants of Rho and Rac1 (RhoA-N19 and Rac1-N17 respectively) in our study. These mutants tagged with CFP were overexpressed in COS-7 cells. At 48 hr post-transfection, the cells were infected with RH strain tachyzoites. Interestingly, the accumulation of these GTPases to the PVM was no longer seen when they were in these inactive forms, which constitutively bind only GDP (Figure 3). Thus, the recruitment of these Rho GTPases to the PVM only occurred when Rho GTPases retained normal activity.

### The Rho A and Rac1 GTPases were activated upon *T. gondii* tachyzoite invasion

**Figure 4:**
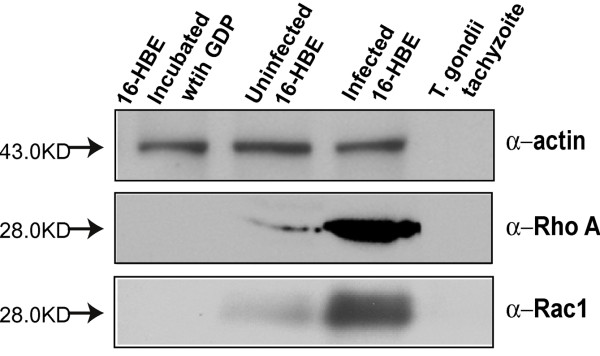
**Detection of RhoA and Rac1 activation in human 16HBE cells following *T. gondii *tachyzoites infection with Rho GST Pull-down assay. ***T. gondii *RH tachyzoites infected human 16-HBE cells and uninfected cells were harvested and lysed. About 150 μg of the total protein from these two cell lysates was used in Rho pulldown assay. GST-tagged Rhotekin-RBD protein on agarose beads for RhoA or GST-tagged PAK-PBD protein bound agarose beads for Rac were used to bind and precipitate only the active form of RhoA or Rac1 in the cell lysate. In the Western-blot, actin was used as the equal protein loading control. The negative control group cell lysate which was pre-incubated with GDP showed no band on the Western-blot membrane. The more intense bands found in the infected cells for anti-RhoA and anti-Rac1 compared to the uninfected cells indicated that more GTP-bound RhoA or Rac1 were precipitated from the infected cell lysate, which were activated upon *T. gondii* invasion.

To determine if RhoA or Rac1 GTPases were actually activated following *T. gondii* tachyzoite invasion, we used GST-tagged Rhotekin-RBD protein on agarose beads specific for RhoA or GST-tagged PAK-PBD protein bound agarose beads specific for Rac only to bind the GTP-bound RhoA or Rac1 in the cell lysate, but not the GDP-bound form. Western-blot analyses detected increased amounts of GTP-bound RhoA and Rac1 from the infected cells compared with the uninfected cells (Figure 4), but no signals were detected in the negative control (16-HBE cells incubated with GDP) or the *T. gondii* infected groups. These results strongly suggest that *T. gondii* invasion results in the activation of RhoA and Rac1 GTPaes.

### The recruitment of RhoA to *T. gondii* PVM is dependent on different RhoA domains

**Figure 5:**
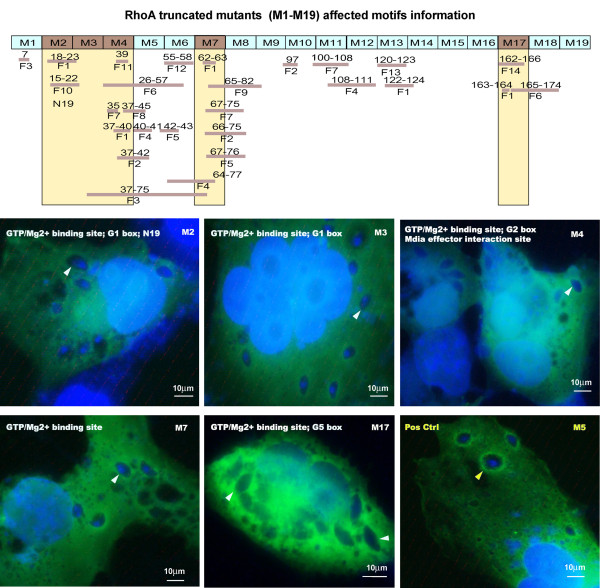
**The recruitment of RhoA to *T. gondii *PVM is dependent on different RhoA domains (1000×). **COS-7 cells were transfected with 3 μg of pEGFP-N1-RhoA mutants’ plasmids M1-M19, respectively. Forty-eight hr post-transfection, the cells were infected with RH strain tachyzoites of *T. gondii*. M2 (RhoA^Δ11–20^), M3 (RhoA^Δ21–30^), M4 (RhoA^Δ31–40^), M7 (RhoA^Δ61–70^) and M17 (RhoA^Δ161–170^) were found not to accumulate on the PVM (white arrowhead and white labeling), indicating that the integrity of the features (**F**) as follows are essential for the recruitment of RhoA to the PVM: F1-GTP/Mg^2+ ^binding site [chemical binding site], F-7:mDia effector interaction site, F-10:G1 box, F-11:G2 box, F-14:G5 box. The other mutants were all equally well recruited to the PVM as RhoA wild-type (yellow arrowhead in M5 is representative, and the other mutants information is provided in Additional file 3: Data S3), indicating that the other motifs of RhoA such as F2-GAP (GTPase-activating protein) interaction site [polypeptide binding site], F3-GEF (guanine nucleotide exchange factor) interaction site [polypeptide binding site], F4-GDI (guanine nucleotide dissociation inhibitor) interaction site [polypeptide binding site], F5-Rho kinase (ROCK) effector interaction site [polypeptide binding site], F6-PKN/PRK1 effector interaction site, F8-Switch I region, F9-Switch II region, F12-G3 box and F13-G4 box, are not the decisive motifs for the recruitment of RhoA to the PVM. Bar: 10 μm.

In order to define what motifs are vital to the recruitment of Rho GTPases to the PVM, we concentrated on the study of Rho A as a representative protein. Sequential deletion of RhoA by 10 amino acids with site-directed mutation from the parental plasmid pECFP-RhoA-WT generated 19 RhoA mutants. The different CFP-tagged, truncated RhoA plasmids (M1-M19) were transfected into COS-7 cells grown on coverslips in 6-well plates and analyzed by immunofluorescence microscopy. M2 (RhoA^Δ11–20^), M3 (RhoA^Δ21–30^), M4 (RhoA^Δ31–40^), M6 (RhoA^Δ51–60^), M17 (RhoA^Δ161–170^) could not be observed on the PVM (Figure 5), indicating the decisive motifs were potentially the GTP/Mg^2+^ binding site, the mDia effector interaction site, the G1 box, the G2 box and the G5 box. The other mutants were all similarly recruited to the PVM as in wild-type RhoA (Additional file 3: Data S3). These results show that the GAP (GTPase-activating protein) interaction site, the GEF (guanine nucleotide exchange factor) interaction site, the GDI (guanine nucleotide dissociation inhibitor) interaction site, the Rho kinase (ROCK) effector interaction site, the PKN/PRK1 effector interaction site, the Switch I region, the Switch II region, the G3 box and the G4 box were not the decisive motifs for the recruitment of RhoA to the PVM.

### The PVM-localized Rho and Rac GTPases do not respond to epithelial growth factor (EGF) activation

**Figure 6:**
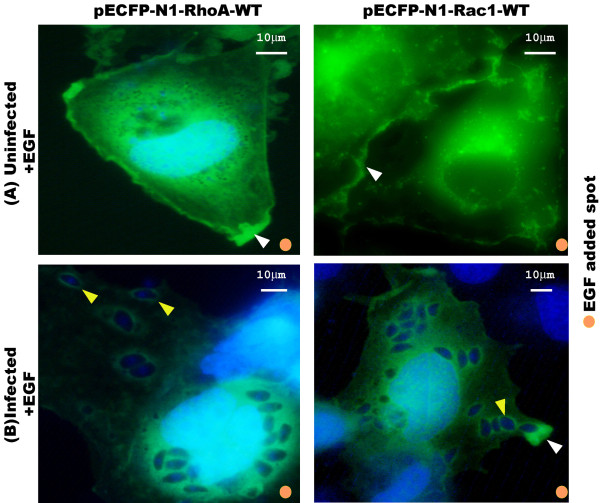
**The CFP-tagged Rho and Rac1 GTPases accumulated on the parasitophorous vacuole membrane (PVM) do not translocate toward epithelial growth factor (EGF) activation.** Two paralleled groups of COS-7 cells were grown on coverslips and transfected with pECFP-RhoA and pECFP-Rac1 respectively. Forty-eight hr post-transfection, cells were starved overnight in serum-free DMEM. One group of cells was infected with *T. gondii* tachyzoites and the other group was kept uninfected. One hr post-infection, the infected cells were washed 3× with PBS to remove the unrecruited tachyzoites. Cells were site-activated with EGF for 5 min. (**A**) In uninfected cells, the CFP-tagged RhoA and Rac1 GTPases in the cytosol translocated to the host cell membrane (white arrowhead) in response to EGF activation. (**B**) In infected cells, the CFP-tagged RhoA and Rac1 were sequestered on the PVM without translocation toward the EGF, while the unassociated RhoA and Rac1 in the cytosol still translocated toward the EGF as in uninfected cells. More photographs provided in Additional file 4: Data S4 showing the RhoA and Rac1 sequestered on the PVM regardless the activation of EGF. Bar: 10 μm.

Rho GTPases control cell motility by regulating the reorganization of the cytoskeleton in response to EGF [17]. Rho and Rac GTPases translocated from the cytosol to the cell membrane upon EGF activation [18]. To study whether the Rho and Rac GTPases accumulated on the PVM would translocate following EGF activation, the COS-7 cells overexpressing CFP-tagged Rho and Rac1 were starved overnight, infected with *T. gondii* RH tachyzoites and then activated with EGF. The result showed that the recruited Rho and Rac GTPases on the PVM did not change in fluorescence brightness, unlike the fluorescence brightness in the cytosol that became faint because of the translocation of RhoA and Rac1 from the cytosol to the cell membrane towards the EGF activation spot (Figure 6). More photographs showing the RhoA and Rac1 sequestered on the PVM regardless the activation of EGF are provided in Additional file 4: Data S4.

### Interference with RhoA and Rac1 endogenous activity affects tachyzoite infection

**Figure 7:**
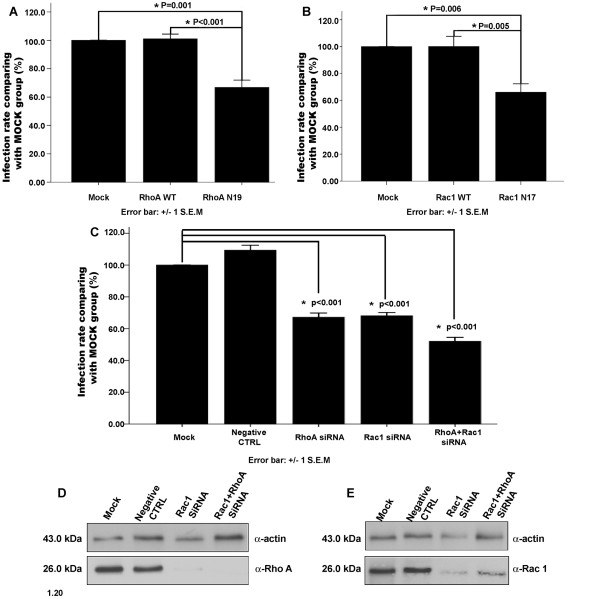
**The overexpression of dominant negative mutants of Rho GTPases and the expression silencing of Rho GTPases in host cells diminished the invasiveness of *T. gondii *RH tachyzoites. **(**A-B**) RhoA or Rac1 overexpression: When compared with the untransfected cells (mock group), RhoA-WT or Rac1-WT overexpressed cells showed the almost same infection rate, while dominant-negative mutant RhoA-N19 or Rac1 N17 overexpressed cells showed a significantly lower infection rate (*P* = 0.001 and *P* = 0.005), proximately 60% of the Mock. (**C**) Silencing of RhoA or Rac1: When compared with the untransfected cells (mock group) and negative control siRNA transfected groups, cells transfected with RhoA siRNA, Rac1 siRNA or RhoA + Rac1 siRNA showed a significantly lower infection rate (*P* < 0.001). It was about 65% of the Mock in the two single knockdown groups and about 50% of the Mock in the double knockdown group. (**D-E**) Detection of RhoA or Rac1 RNAi efficiency: anti-actin panel showed the same amount of total protein was loaded for detection in different cell lysates including mock, negative control siRNA, RhoA or RAC1 siRNA, and RhoA + Rac1 siRNA transfected groups. Anti-RhoA panel showed the apparent inhibition of RhoA expression in RhoA silenced and RhoA + Rac1 silenced cells; anti-Rac1 panel showed the apparent inhibition of Rac1 expression in Rac1 and RhoA + Rac1 silenced cells.

To study the role of host cell RhoA and Rac1 GTPases during the tachyzoites invasion, COS-7 cells were over-expressed with RhoA-WT, RhoA-N19, Rac1-WT, and Rac1-N17. The endogenous expression of RhoA or Rac1 was inhibited by siRNA targeted towards either RhoA or Rac1 separately or towards both in human 16-HBE cells and then infected with RH tachyzoites. The infection rate was determined for each group. The results indicated that no significant difference was found between the infection rates of RhoA-WT or Rac1-WT overexpressed cells and the mock cells (Figure 7A-B, respectively). However, the dominant negative mutants RhoA-N19 and Rac1-N17 overexpressed in COS-7 cells inhibited the cell invasion by *T. gondii* tachyzoites significantly; the infection rates were approximately 60% of that of the mock cells (p < 0.01) (Figure 7A-B, respectively). Silencing RhoA, Rac1 or both RhoA and Rac1 in 16-HBE cells also showed a significant inhibition of cell invasion by tachyzoites (p < 0.01) Figure 7C-E). The infection rates of RhoA and Rac1 silenced cells were about 65% of that of the mock cells, while the infection rate of RhoA and Rac2 double-silenced cells was about 50% of that of the mock cells (Figure 7C).

## Discussion

### The function of the Rho and Rac GTPases accumulated on PVM

Immunity-related GTPases (IRGs) also known as p47 GTPases, are key mediators of interferon-gamma-induced resistance to pathogens [19]. They cycle between GDP-GTP bound forms, and cooperatively oligomerize in the GTP-bound conformation on the *T. gondii* PVM [20]. Sequential recruitment of multiple IRGs to the PVM results in disruption of PVM and parasite digestion within 2 hr of infection [21]. Virulent type I strains resist recruitment and avoid clearance, while less virulent type II and III strains are effectively cleared by IRGs [22]. It was reported that a serine threonine kinase secreted by *T. gondii,* ROP18, binds to and phosphorylates IRGs on the PVM, and the phosphorylation of IRGs prevented clearance of *T. gondii* within inflammatory monocytes and IFN-γ-activated macrophages, conferring parasite survival *in vivo*[23]. ARF6 is a small GTPase of the ARF family that regulates membrane trafficking and actin cytoskeleton rearrangements at the plasma membrane. ARF6 was found recruited to the PV of *T. gondii* tachyzoites and ARF6 activity was necessary for cell invasion by tachyzoites of *T. gondii*[14]. These reports about the function of the GTPases on the PVM in *T. gondii* invasion urged us to hypothesize what is the function of the host cell Rho and Rac1 accumulating on the PVM.

Both the indirect immunofluorescence staining of the endogenous RhoA and Rac1 of the host cell, and the over-expressed CFP-tagged RhoA and Rac1 recombinant proteins in the host cell indicated the recruitment of RhoA and Rac1 in the PVM of *T. gondii* tachyzoites (Figure 1). From the real-time observation of the invasion of the host cell by *T. gondii* tachyzoites, we found that the recruitment of RhoA to the PVM happened at the very beginning of the invasion either from the membrane or from the cytosol (Figure 2). Those over-expressed CFP-tagged dominant negative mutants RhoA-N19 and Rac1-N17 did not accumulate to the PVM (Figure 3) implying the recruitment of RhoA and Rac1 is dependent on their GTPase activity. The GST-pull down assay detected greater amounts of GTP-bound RhoA and Rac1 in the infected host cells than in uninfected cells (Figure 4). Through CFP-tagged RhoA and Rac1 being visualized under the GFP filter, we found that RhoA and Rac1 GTPases in the host cell cytosol were translocated to the host cell membrane following EGF activation, while unlike the GTPases in the cytosol, RhoA or Rac1 on the PVM did not diffuse, translocate or respond to EGF activation. EGF activates RhoA and Rac1 through activation of the EGF pathway [24,25]. This observation led us to hypothesize that the Rho and Rac1 GTPase recruited on the PVM probably was GTP-bound and could not be activated again by EGF, while most of the GTPases in the cytosol are in GDP-bound form and could be continually activated and translocated to the cell membrane upon EGF activation (Figure 6).

These observed results imply the invasion of the tachyzoites need the activation of RhoA and Rac1 GTPases; and the recruitment of these activated GTPases to the PVM is much more than a phenomenon as it may perform some as yet undefined but important function(s).

### The decisive RhoA GTPases motifs for recruitment to parasitophorous vacuole membrane following *T. gondii* invasion

Wild-type Rho and Rac GTPases with normal GTPase activity were recruited to the PVM, but those mutants that constitutively bind only GDP (RhoA-N19 and Rac1-N17) lacked this ability. The 10 amino acid sequentially deleted RhoA mutants were used in the identification of the definitive motif(s) necessary for the recruitment to the PVM. M2 (RhoA^Δ11–20^), M3 (RhoA^Δ21–30^), M4 (RhoA^Δ31–40^), M7 (RhoA^Δ61–70^) and M17 (RhoA^Δ161–170^) lacked the ability to be recruited to the PVM (Figure 5). Based on the analysis of the RhoA GTPase conserved domains[26], the GTP/Mg^2+^ binding site, the mDia effector interaction site, the G1 box, the G2 box and the G5 box are the most potential motifs that determine the recruitment of RhoA GTPases to the PVM (Figure 5).

Rho GTPases are molecular switches that cycle between an active GTP-bound and an inactive GDP-bound form, which regulate many essential cellular processes, including actin dynamics, gene transcription, cell-cycle progression and cell adhesion [27]. When in the active forms, Rho GTPases are able to interact with effector or target molecules to initiate downstream responses, signal transduction terminates when GTP is hydrolyzed to form GDP, and at which point the cycle is finished completely [27]. The GTP/Mg^2+^ binding site of Rho GTPases is used to bind GTP and Mg^2+^, which activates the GTPases [28]. The mDia effector interaction site is the domain that binds with mDia as a downstream Rho effector involved in microtubule stabilization. The mDia site induces stable microtubules that are capped and indicates that mDia may promote this microtubule capping by directly binding to microtubules. [29]. The G1-G5 boxes are the GDP/GTP-binding motif elements that comprise a ~ 20 kDa phosphate domain (G domain, Ras residues 5–166), which is conserved in all Ras super family proteins [30]. The decisive motifs are either related to GTP binding or with the effector regulating microtubules. This finding is consistent with our proposal that the recruitment of Rho GTPase to PVM depends on its enzymatic activity, and the invasion of *T. gondii* needs the rearrangement of host cell cytoskeleton.

### Host cell RhoA and Rac1 activation is required for efficient cell invasion by *T. gondii* tachyzoites, which is a shared mechanism by many other intracellular pathogens infection

The major function of Rho GTPases activation is to regulate the dynamics and organization of the actin cytoskeleton [17], which is vital to the cell invasion of *T. gondii* tachyzoites. First, *T. gondii* tachyzoites invasion activates the reorganization of the microfilaments and microtubules of the host cell [31,32]. Reorganization of host cell F-actin during entry of *Toxoplasma* tachyzoites has been visualized, and the entry was dependent on the actin dynamics [31]. Second, any treatment to cease the normal cytoskeleton reorganization of host cells will impair *T. gondii* invasion efficiency. Cell invasion by *T. gondii* tachyzoites is significantly inhibited in cells treated with colchicum (a MT inhibitor) [33], cytochalasin D (an actin inhibitor) [14,33] and jasplakinolide (a chemical disrupting actin filaments, which induces actin polymerization) [31]. Maintenance of host cell actin cytoskeleton integrity is important to parasite invasion [14].

In our research, no significant difference was found in the infection rates of *T. gondii* tachyzoites in the cells overexpressed with RhoA or Rac1 wild-type proteins compared with those untransfected cells, while either overexpression of dominant negative mutants of RhoA or Rac1 or inhibition of endogenous RhoA or Rac1 in the host cell could significantly reduce cell invasion (Figure 7). This result indicated that it is the normal endogenous activity of RhoA and Rac1 that defines the efficiency of cell invasion by *T. gondii* tachyzoites, but not the amount of these proteins. This requirement is also reported in other intracellular pathogens. *Shigella* entry into HeLa cells induces membrane ruffling at the bacterial entry site, and the three Rho isoforms were recruited into bacterial entry sites. This membrane folding caused by invasion was abolished by using a Rho-specific inhibitor, and bacterial entry was impaired accordingly [34]. Hela cells transfected with the dominant negative versions of Rac1 or RhoA reduced group B *Streptococcus* invasion by 75% and 51%, respectively, suggesting that Rho GTPases are indispensable for efficient invasion of HeLa cells by this bacterium [35]. In MDCK cells, RhoA and Rac1were activated during *Trypanosoma cruzi* invasion and then triggered the reorganization of F-actin cytoskeleton, especially distinct in the invasion position on the cell membrane. The invasion of *T. cruzi* G strain extracellular amastigotes was specifically inhibited in Rac1-N17 dominant-negative cells [36,37]. After the invasion of the rabbit corneal epithelial cells (SIRC) by *Candida albicans*, host cell actin filaments formed a rigid ring-like structure in the host cell. Immunochemical staining of actin and the expression of chimeric green fluorescent protein (GFP)-GTPases (RhoA, Rac1) showed the colocalization of the GTPases with actin at invasion and actin polymerization sites, but this colocalization was not seen in SIRC cells expressing a GFP-tagged dominant-negative mutant of GTPases. Inhibition of invasion was observed in SIRC cells expressing dominant-negative mutants of Rac1 and RhoA GTPases [38]. These findings suggest that many pathogens may employ conserved pathways for invasion.

### The Rho and Rac cell signaling involved in the cytoskeleton reorganization triggered by *T. gondii* invasion

**Figure 8:**
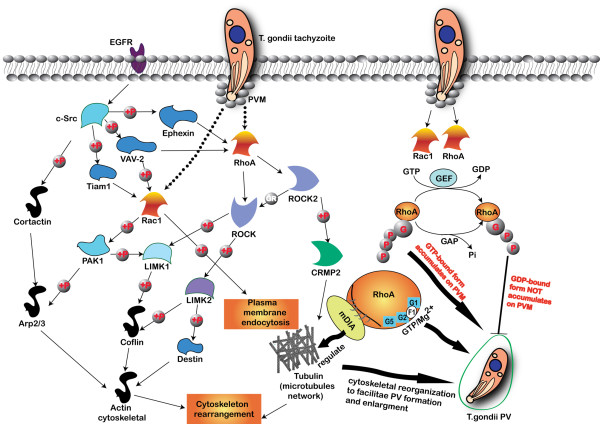
**Cell signaling related to RhoA and Rac1 regulated cytoskeleton reorganization in *T. gondii *infection. **c-Src is activated by EGF induced EGF receptor activation and followed by Ephexin, VAV-2 and Tiam 1 phosphorylation. Ephexin phosphorylation promotes its GTPase activity toward RhoA and ROCK. ROCK directly phosphorylates LIMK1 and LIMK2, which in turn phosphorylate destrin and cofilin. ROCK2 phosphorylates CRMP2, and CRMP2 phosphorylation reduces its tubulin-heterodimer binding and the promotion of microtubule assembly. Activation of VAV-2 activates RhoA and Rac1. In the downstream of Rac1, p21-activated kinase 1 (PAK1) activates LIMK1 and regulates the actin cytoskeletal reorganization through the phosphorylation of the actin-depolymerizing factor cofilin and destrin. PAK1 also phosphorylates Arp2/3 complex to promote actin polymerization. Cortactin is a prominent target of c-Src, and regulates cytoskeletal dynamics. Tyrosine phosphorylation of cortactin reduces its F-actin cross-linking capability. In our research, we are not clear about the upstream of the RhoA and Rac1 GTPases cell signaling involved in *T. gondii *infection, but we can see the activation of RhoA and Rac1 of host cells and the reorganization of the cytoskeleton for PV formation. RhoA and Rac1 GTPases accumulate on the PMV regardless of the parasitic strain virulence, and the accumulation is dependent on their GTPase activity. The recruited RhoA or Rac1 on the PVM are probably in GTP-bound active form. The RhoA GTPase is recruited to the PVM as soon as the T. gondii tachyzoite invaded the host cell either through the host cell membrane or from the cytosol. The decisive domains for the RhoA accumulation on the PVM includes the GTP/Mg2+ binding site (F1), the mDia effector interaction site, the G1 box (G1), the G2 box (G2) and the G5 box (G5). The reorganization of host cell cytoskeleton facilitates the formation and enlargement of *T. gondii *PV in the host cell.

When epithelial cells are stimulated by EGF, c-Src is activated by EGF-induced EGF receptor activation [39]. After the activation of c-Src, Ephexin, VAV-2 and Tiam 1 are rapidly phosphorylated by c-Src [40,41]. Phosphorylation of Ephexin promotes its GTPase activity toward RhoA [42,43], and RhoA downstream effector Rho-associated kinase ROCK directly phosphorylates LIM-kinases LIMK1 and LIMK2, which in turn phosphorylates actin-depolymerizing factor destrin and actin-associated protein cofilin [44]. ROCK2 kinase phosphorylates CRMP2, and the phosphorylation of CRMP2 reduces its tubulin-heterodimer binding and the promotion of microtubule assembly [45,46]. Activation of VAV-2 activates RhoA and Rac1 [47]. Downstream of Rac1, p21-activated kinase 1 (PAK1) activates LIMK1, and regulates the actin cytoskeletal reorganization through the phosphorylation of the actin-depolymerizing factors cofilin and destrin and their actin-depolymerizing activities [48,49]. PAK1 also phosphorylates Arp2/3 complex to promote actin polymerization [50]. The F-actin-binding protein cortactin is a prominent target of various tyrosine kinases (c-Src) and regulates cytoskeletal dynamics [42,50]. Tyrosine phosphorylation of cortactin has been suggested to reduce its F-actin cross-linking capability [51]. In our research, we are not clear about the upstream cell signaling component of the Rho and Rac GTPases involved in *T. gondii* infection, but we have witnessed the activation of RhoA and Rac1 of host cells and the reorganization of the cytoskeleton for PV formation during the infection of *T. gondii*. The cell signaling involved in this process is shown in Figure 8.

## Conclusion

RhoA and Rac1 GTPases from the host cell accumulated on the PVM after *T. gondii* invasion, and this accumulation was dependent on their GTPase activity and occurred regardless of the virulence of the parasitic strain. RhoA GTPase was recruited to the PVM as soon as the *T. gondii* tachyzoite invaded the host cell either through the host cell membrane or from the cytosol. Host cell RhoA and Rac1 were activated after *T. gondii* invasion. The decisive domains for the RhoA accumulation on the PVM were identified as the GTP/Mg^2+^ binding site, the mDia effector interaction site, the G1 box, the G2 box and the G5 box, respectively, which were related to the binding of GTP for enzymatic activity and to mDia for the regulation of microtubules. The reorganization of host cell cytoskeleton facilitates the PV formation and enlargement in the host cell. The recruited RhoA on the PVM could not be activated by epithelial growth factor (EGF) and no translocation was observed, which indicated that the recruited RhoA or Rac1 on the PVM might be in GTP-bound active form.

Wild-type RhoA or Rac1 overexpressed cells had almost the same infection rates by *T. gondii* as the mock-treated cells, while RhoA-N19 or Rac1-N17 transfected cells and RhoA or Rac1 siRNA- and RhoA + Rac1 siRNA-treated cells showed significantly diminished infection rates than mock cells, which indicated that the normal activity of RhoA and Rac1 GTPases are indispensable to the internalization of the tachyzoite. The accumulation of the RhoA and Rac1 on the PVM and the requisite of their normal GTPase activities for efficient invasion implied their involvement and function in *T. gondii* invasion. The summary of the host cell RhoA and Rac1 cell signaling involved in the *T. gondii* invasion is show in Figure 8.

## Abbreviations

PV: Parasitophorous vacuole; PVM: Parasitophorous vacuole membrane; MJ: Moving junction; EGF: Epithelial growth factor; IRG: Immunity-related GTPase; Arf 6: ADP-ribosylation factor-6.

## Competing interests

The authors declare that they have no competing interests

## Authors’ contributions

R-HN: cell culture, GST-pull down assay, fluorescence microscopy. G-HZ: site directed mutation, fluorescence microscopy. J-XL: T. gondii infection. X-JM: Real-time photography. LC: manuscript revising and suggestion. H-JP: conception and design, supervision of the research group, funding support, drafting the manuscript. X-gC: analysis and interpretation of data funding support. JG-C: manuscript revising and suggestion. All authors read and approved the final manuscript.

## Supplementary Material

Additional file 1 **Data S1. **The florescence images of the real-time observation of the cell invasion by *T. gondii*. The invasion position was indicated with a purple arrowhead. The green florescence pictures showed the accumulation of the CFP-tagged RhoA to the PVM (purple arrowhead) at the time points of -10 min (5 min post infection), -5 min (10 min post infection), 0 min (15 min post infection), 5 min (20 min post infection), 10 min (25 min post infection) and 15 min (30 min post infection). The focal point of RhoA at the immediate point of invasion on the host cell membrane is not visible. Click here for file

Additional file 2 **Data S2. **The DIC images of the real-time observation of the cell invasion by *T. gondii*. For the convenience of recognizing the invasion position, the DIC images were overlaid with the fluorescence images. The three tachyzoites invading the host cell are shown in white, yellow and purple arrowheads, respectively. Starting from 5 min post infection, the invasion of tachyzoites into the host cell was visualized and pictures were taken at 10 min intervals. Refer to the legends of Figure 2. Click here for file

Additional file 3 **Data S3. **The unessential motif truncated mutants of RhoA accumulating on the PVM. The COS-7 cells were transfected with the plasmids of CFP-tagged M1, M5, M6, M8, M9, M10, M11, M12, M13, M14, M15, M16, M18 and M19 truncated RhoA, and 48 hr post-transfection, the cells were infected with tachyzoites of RH strain. The recruitment of these CFP-tagged mutants on the PVM was visualized using a fluorescence microscope. Click here for file

Additional file 4 **Data S4. **The CFP-tagged Rho and Rac1 GTPases accumulated on the parasitophorous vacuole membrane (PVM) do not translocate toward epithelial growth factor (EGF) activation (more data). Yellow arrowhead indicates the CFP-tagged RhoA/Rac1 GTPases accumulated on the PVM (no translocation following EGF activation). White arrowhead indicates the translocated RhoA to the host cell membrane ruffling towards EGF activation. Click here for file
