# Supplementary figures and images for: Enzymatically active Rho and Rac small-GTPases are involved in the establishment of the vacuolar membrane after Toxoplasma gondii invasion of host cells
Source: BMC Microbiol. 2013 May 30;13:125. doi: 10.1186/1471-2180-13-125 (PMC3681593; doi:10.1186/1471-2180-13-125)

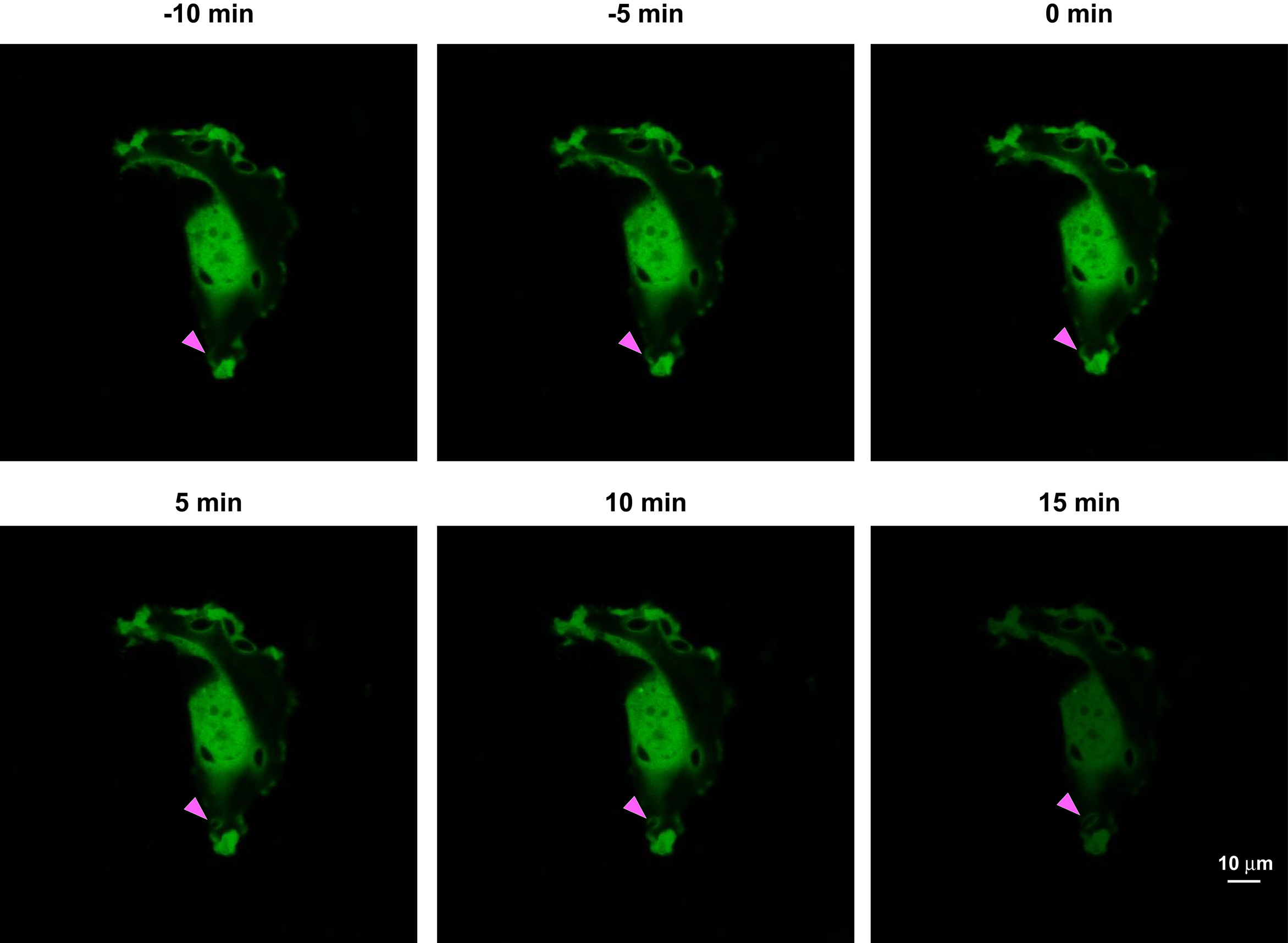

Supplement: Additional file 1 — Data S1. The florescence images of the real-time observation of the cell invasion by T. gondii. The invasion position was indicated with a purple arrowhead. The green florescence pictures showed the accumulation of the CFP-tagged RhoA to the PVM (purple arrowhead) at the time points of -10 min (5 min post infection), -5 min (10 min post infection), 0 min (15 min post infection), 5 min (20 min post infection), 10 min (25 min post infection) and 15 min (30 min post infection). The focal point of RhoA at the immediate point of invasion on the host cell membrane is not visible. [file 1471-2180-13-125-S1.jpg]

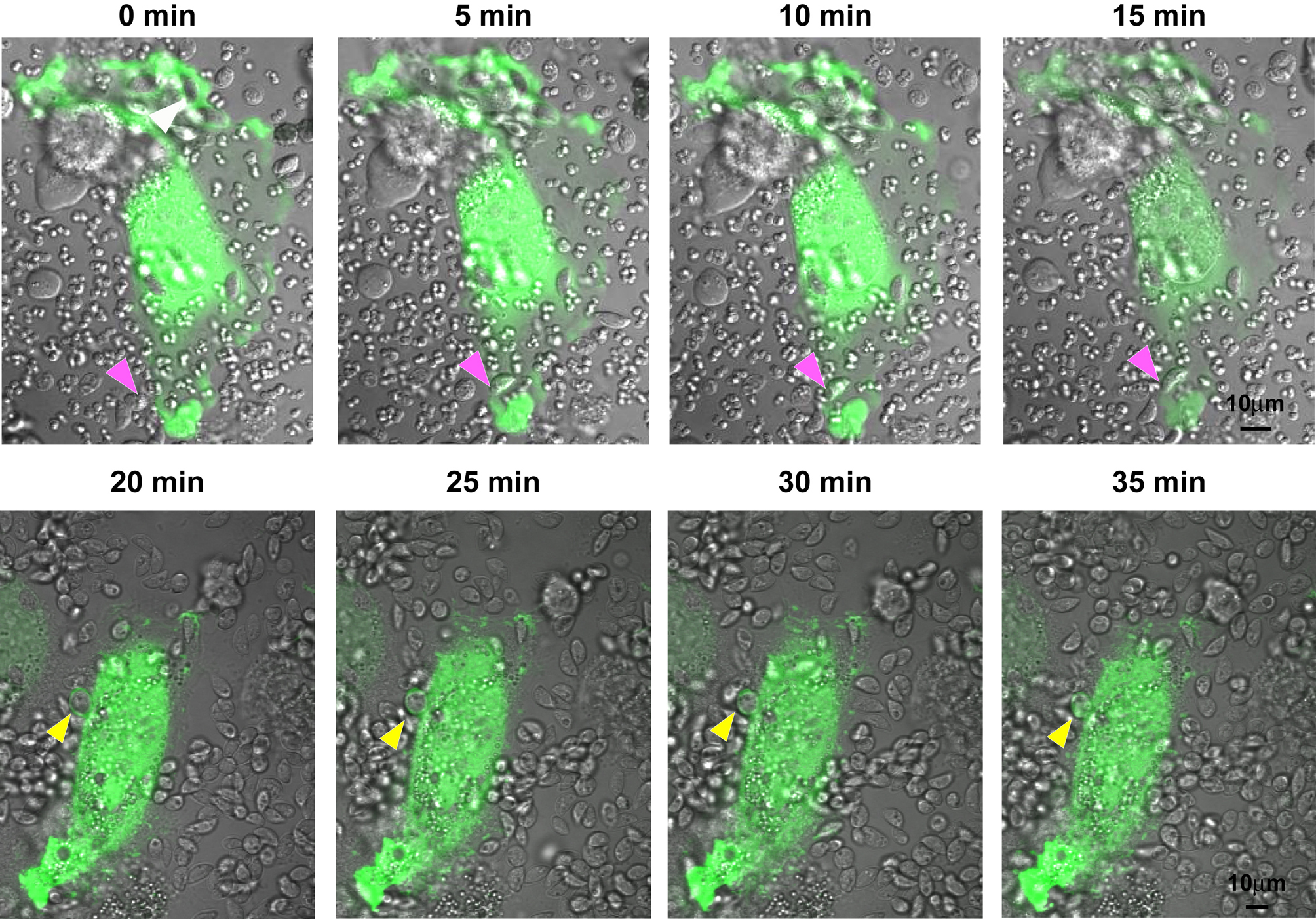

Supplement: Additional file 2 — Data S2. The DIC images of the real-time observation of the cell invasion by T. gondii. For the convenience of recognizing the invasion position, the DIC images were overlaid with the fluorescence images. The three tachyzoites invading the host cell are shown in white, yellow and purple arrowheads, respectively. Starting from 5 min post infection, the invasion of tachyzoites into the host cell was visualized and pictures were taken at 10 min intervals. Refer to the legends of Figure 2. [file 1471-2180-13-125-S2.jpeg]

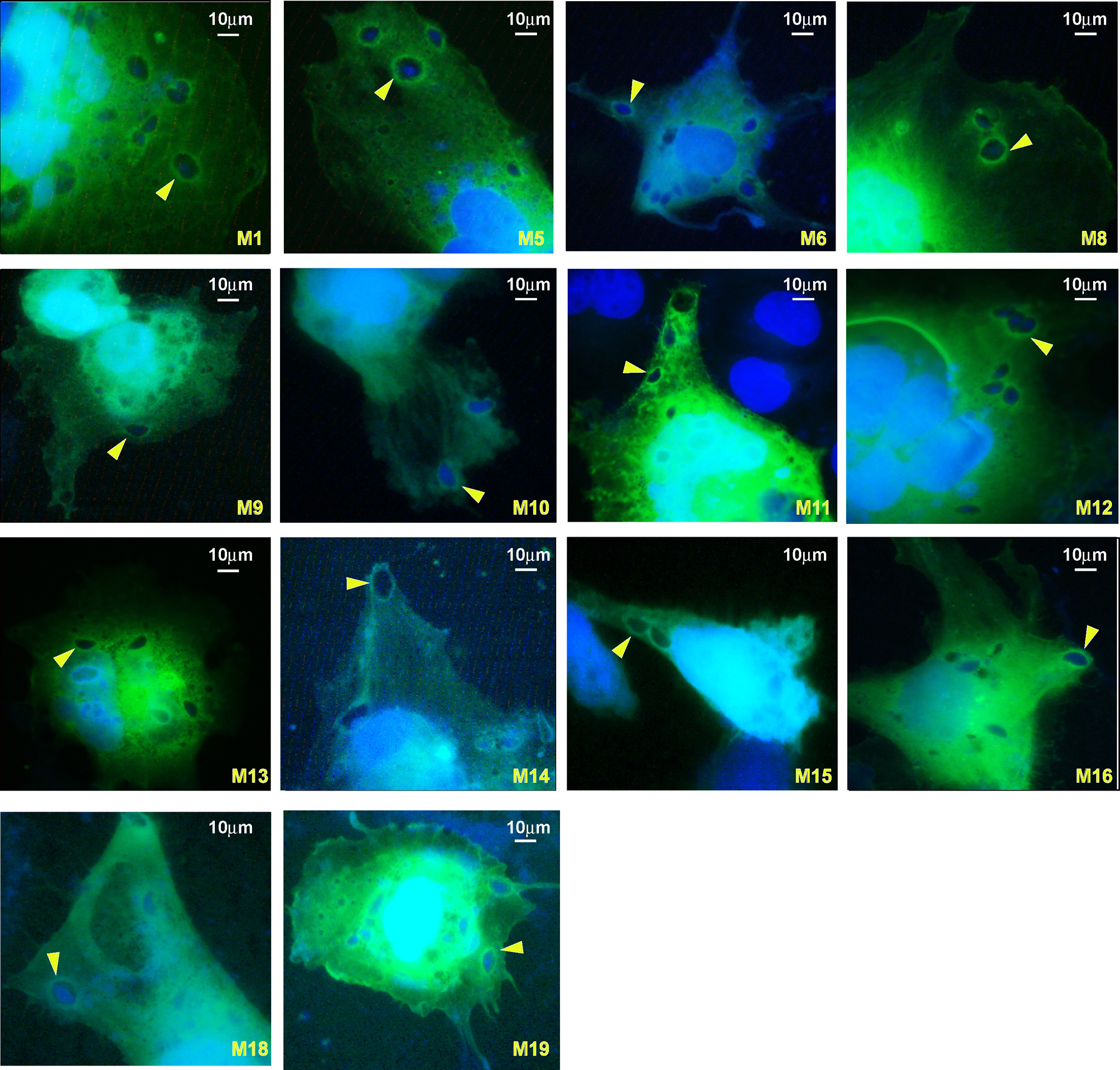

Supplement: Additional file 3 — Data S3. The unessential motif truncated mutants of RhoA accumulating on the PVM. The COS-7 cells were transfected with the plasmids of CFP-tagged M1, M5, M6, M8, M9, M10, M11, M12, M13, M14, M15, M16, M18 and M19 truncated RhoA, and 48 hr post-transfection, the cells were infected with tachyzoites of RH strain. The recruitment of these CFP-tagged mutants on the PVM was visualized using a fluorescence microscope. [file 1471-2180-13-125-S3.jpeg]

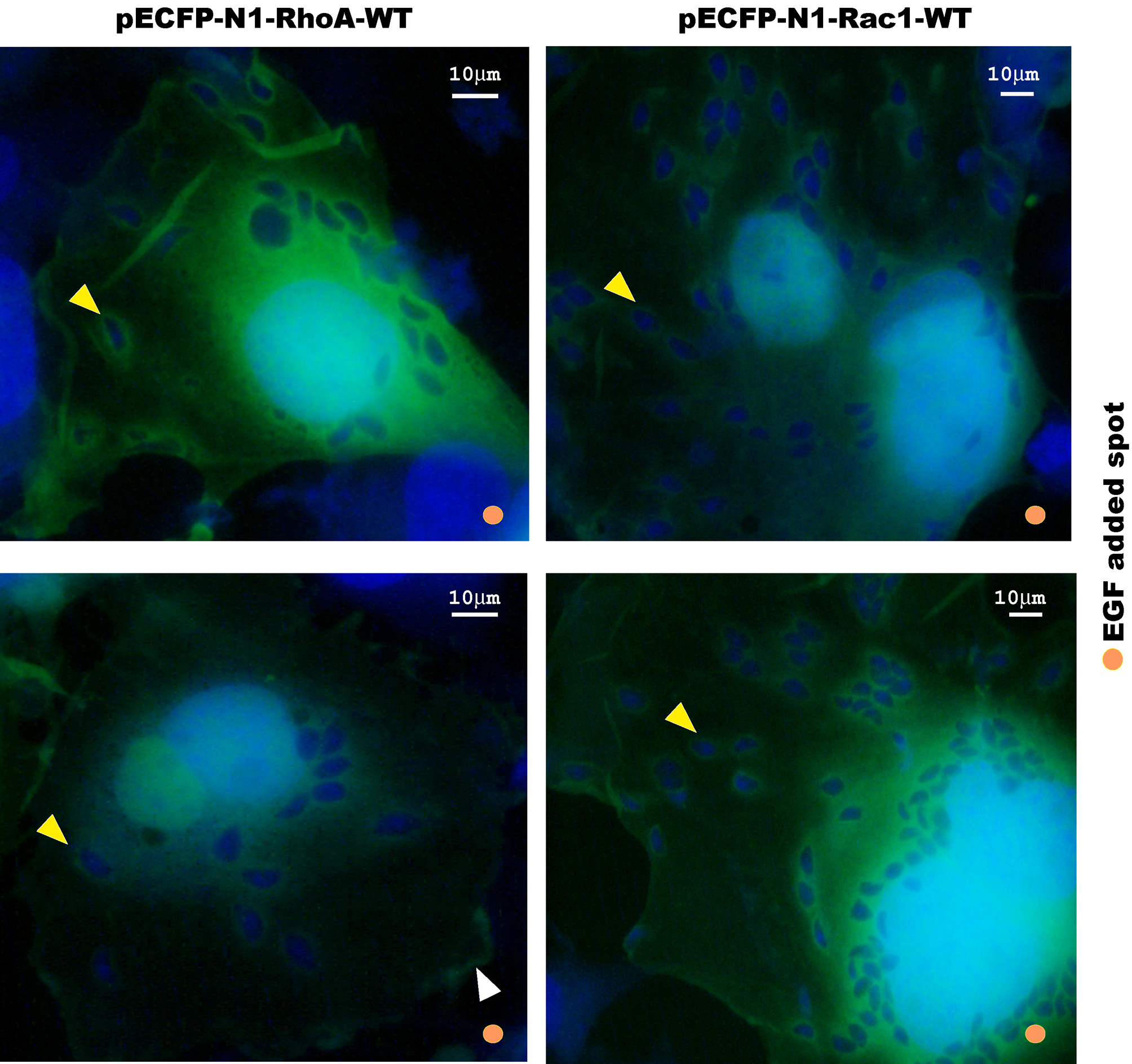

Supplement: Additional file 4 — Data S4. The CFP-tagged Rho and Rac1 GTPases accumulated on the parasitophorous vacuole membrane (PVM) do not translocate toward epithelial growth factor (EGF) activation (more data). Yellow arrowhead indicates the CFP-tagged RhoA/Rac1 GTPases accumulated on the PVM (no translocation following EGF activation). White arrowhead indicates the translocated RhoA to the host cell membrane ruffling towards EGF activation. [file 1471-2180-13-125-S4.jpeg]
